# Effect of doctor allocation policies on the equitable distribution of doctors in Thailand

**DOI:** 10.1186/s12960-022-00782-5

**Published:** 2023-01-13

**Authors:** Thinakorn Noree, Nonglak Pagaiya, Intira Nimnual

**Affiliations:** 1 International Health Policy Program, Ministry of Public Health, Tiwanon Road, Muang, 11000 Nothaburi Thailand; 2 Faculty of Public Health, Khonkaen University, 123 Mitraphap Road, Muang, Khonkaen, 40000 Thailand

**Keywords:** Geographic distribution, Doctors, Gini coefficient

## Abstract

**Background:**

Equitable geographic distribution of doctors is crucial for the provision of an accessible and efficient health service system. This study aimed to assess the effects of doctor allocation by the Thai Ministry of Public Health (MoPH) in relation to equity distribution.

**Methods:**

This descriptive study compared secondary data on the number of doctors, gross provincial products (GPP), and populations of 76 Thai provinces for the years 2017 and 2021. The ratio of doctors to 1000 population was used to measure the spatial distribution of doctors by province. Lorenz curves and the Gini coefficient were used to determine the equity of doctor distribution.

**Results:**

The results showed that the Gini coefficient decreased from 0.191 in 2017 to 0.03 in 2021 indicating that the equitable distribution of doctors improved after the MoPH commenced allocating newly graduated doctors according to health utilization in 2017. Compared to 2017, the percentage changes in the number of doctors were higher in provinces with lower doctor densities and in provinces with higher GPPs.

**Conclusion:**

The equitable distribution of doctors in Thailand was affected by two main causes: the allocation of newly graduated doctors by the MoPH and the turnover rate of existing doctors.

## Background

Ensuring an adequate supply and equitable distribution of health workers is of high importance to achieving effective universal health coverage and sustainable development goals. The equitable geographical distribution of doctors is a recurring global health workforce challenge and has been part of the health policy discussion for many decades. Studies have shown that higher densities of doctors are associated with better health outcomes such as increased life expectancies and decreased standardized death rates [1–5]. The concentration of doctors in one region at the expense of other regions, such as the high concentrations of doctors in large cities and urban areas, has led to inequitable access to health care in many countries [6]. Furthermore, socio-cultural changes and the increasing incidence of chronic conditions in aging populations are increasing demand for health care much faster than the supply of doctors [1], which effectively widens these inequitable workforce distribution gaps.

Thailand is an upper middle-income country that implemented a universal health coverage scheme in 2002, and by 2017, 99.84% of the Thai population had some form of health coverage [7]. Available health facilities delivering services to people can be classified according to a three-tiered service system composed of primary, secondary and tertiary care facilities. Primary health care facilities provide services that incorporate common illness treatment, health promotion, disease prevention, rehabilitation and community health interventions. This type of health facility includes health centers, which are mostly under the control of the Ministry of Public Health (MoPH). Secondary health care facilities provide curative care following referral from primary care facilities. Such health facilities include the MoPH administered district hospitals, other public hospitals, and private hospitals. Tertiary health care facilities provide specialized care, usually after referral from primary or secondary health care facilities. These facilities include MoPH administered general and regional hospitals, university hospitals, and other public hospitals and private hospitals [7]. Many health facilities are concentrated in the capital Bangkok, which is home to 5,487,876 people. Of the public hospitals in Bangkok in 2017, 5 were medical school hospitals, 18 were specialist hospitals, 26 were general hospitals and 137 were medical centers. In addition, 108 of the nation’s 308 private hospitals (35%) were located in Bangkok in 2017 [7]. At the regional level, which covers the 76 provinces excluding Bangkok, there were 6 medical school hospitals, 49 specialist hospitals and 28 regional hospitals in 2017. At the provincial level, health facilities comprised general or regional hospitals, district hospitals and health centers. In 2017, there were 88 general hospitals covering all provinces, 780 district hospitals covering 88.8% of districts, and 9,777 health centers, functioning as primary health care facilities, covering all sub-districts [7]. Almost all health facilities at provincial level are under the responsibility of the MoPH, but private hospitals were also found in big regional centers.

The number of doctors, including general practitioners and all specialist doctors, was 41,746 in 2013 and the ratio of doctors per 1,000 population was 0.65 [8]. The annual production of general practitioners from all medical schools in Thailand has increased from 1676 in 2013 to 3218 in 2017 [7], but the density of doctors in Thailand remains well below the average doctor density in South East Asian countries (1.1/1000 population) and Europe (2.9/1000 population) [9], indicating a shortages of doctors. In addition, doctors in Thailand are not equitably distributed between rural and urban areas or between provinces with low economic status and wealthier provinces [4], hindering the effective provision of health care services by the Thai health system. The Thai government has implemented a multi-pronged intervention strategy over decades to attract and retain doctors in underserved areas. To increase the number of doctors serving rural areas, a special track for student recruitment and training has been implemented that enrolls students with rural backgrounds, provides training at medical schools and MoPH hospitals close to their hometowns, and obliges them to return to their home provinces upon graduation. These interventions help increase the likelihood of medical graduates choosing to work and remain in rural areas [10–12]. This special track currently accounts for 47% of the total number of new graduates (general doctors) employed to work in MoPH facilities annually [10, 11]. In additional, financial and non-financial incentives have been implemented to attract and retain doctors to MoPH facilities, details of which can be seen in the reviews of Wibulpolprasert el al [13] and Pagaiya and Noree [14]. However, as the overall health needs of the population continue to increase due to the increasing proportion of elderly patients and the increasing incidence of chronic diseases, more health workers are required. The current situation combines an overall shortage of doctors with increased demand, which has further aggravated the maldistribution of doctors.

In a strategy to address doctor maldistribution, the MoPH began allocating newly graduated general practitioners according to health service utilization in 2017 [15]. By this approach, the doctor requirements of each hospital in 2017 were determined based on service utilization in that hospital in 2016. The services were outpatient visits, inpatient days, operation services, delivery services, and accident and emergency services. In addition, at each hospital, a ratio of one doctor for five health centers was used to estimate the number of doctors required to serve primary health care, and the ratio of doctor per health centers were based on the service utilization at health center facilities [15]. The total number of doctors required for all MoPH facilities in 2017–2021 was forecast to be 20,053–24,668 doctors. To achieve this target, newly graduated general practitioners were allocated to each province during 2017–2021 according to service utilization and number of health centers in the area. It is important that doctor distribution is equitable, so that people receive accessible, qualified and efficient health services. Therefore, this study aimed to assess the effects of doctor allocation by the MoPH in relation to equity distribution.

## Methods

This descriptive study used secondary data on the number of doctors, gross provincial product (GPP), and population in the years 2017 and 2021. Aggregated data of doctors working at all regional hospitals, general hospitals and district hospitals in 2017 and 2021 were originally obtained from the MoPH administrative information system [16], which is updated annually. The data were segregated by province. GPP by province was derived from the annual report of the office of the National Economic and Social Development Council (NESDC) [17, 18]. The NESDC annually compiles the country gross domestic product (GDP) and then disaggregates this into gross regional products (GRP) and GPP. As the GPP for the year 2021 was not yet compiled, the GPP in 2020 was used. Population data by province were based the National Statistical Office. Population data are collected annually by the Provincial Statistical Office [17, 18]. Population by province in the years 2017 and 2021 was used to calculate the doctor-to-population ratio.

The units of analysis were the 76 provinces excluding Bangkok. Bangkok was excluded from the study as there is a large proportion of care providers in Bangkok that are not covered by the MoPH data system, and the aggregate doctor data in the years 2017 and 2021 were not available. Doctor density is much higher in Bangkok than the overall country ratio (1.59 doctors per 1000 population versus 0.54 doctor per 1000 population in 2016) [7], so including Bangkok’s doctor data would skew the dataset.

### Main outcomes and statistical analysis

To measure the equitable distribution of doctors, the doctor-to-population ratio was used by province to measure the spatial distribution. Unequal geographic distribution means that the spatial distribution of doctors does not match the spatial distribution of the general population. Doctor to population ratios were computed counting the number of doctors working under MoPH health facilities in each province in 2017 and 2021 and the population counts of each province in the same years. The ratios were computed and are expressed as number of doctors per 1000 population for each province. As the MoPH is responsible for the majority of health facilities at provincial level, the doctor distribution data of the MoPH were used to assess the doctor-to-population ratio.

Lorenz curves and an associated measure, the Gini concentration index, were used to determine the equity of the distribution of doctors. The Gini coefficient has been used as the equity indicator in several similar analyses [2, 3, 19]. A Lorenz curve is a cumulative frequency curve that compares the distribution of doctor-to-population ratios to a uniform distribution that represents equality. The Gini coefficient measures the degree of departure from the uniform distribution of the Lorenz curve and takes a value between 0 (indicating perfect equality) and 1 (indicating perfect inequality). In this study, Lorenz curves were plotted based on the cumulative proportions of the doctor-to-population ratio and the cumulative proportions of provincial incomes ranked by GPP per capita. The Gini coefficient (G) was calculated based on the following formula:

$$G = 1 - \sum\limits_{n = 0}^{n - 1} {(Y_{i + 1} + Y_{i} )(} X_{i + 1} - X_{i} ),$$

where n: total number of provinces, Y_i_: cumulative proportions of the doctor-to-population ratio in the *i*th province, and X_i_: cumulative proportions of the GPP per capita in the *i*th province.

Changes in doctor-to-population ratios between 2017 and 2021 were analyzed in this study. Two groups were analyzed. The first group contained the doctor-to-population ratios in 2017. Provinces were categorized based on the average and standard deviation (SD) of the doctor-to-population ratio according to 4 sub-groups based on the average plus and minus 1 and 2 standard deviation(s). The second group was divided by GPP level, as several studies have demonstrated that the supply of health professionals shows a close relationship with gross domestic product (GDP) per capita and income [2, 4, 6]. The provincial GPPs in 2017 were used to categorize the 76 provinces into quartiles. The provinces in the first quartile were specified as having the lowest GPP and the provinces in the fourth quartile were categorized as having the highest GPP. Then changes in the doctor-to-population ratios of each sub-group between 2017 and 2021 were analyzed. The change of Gini coefficient between 2017 and 2021 was also computed and compared.

### Ethics approval

The study used secondary data from open public resources, so no ethics approval was required.

## Results

In 2017, a total of 17,072 doctors were working at regional hospitals, general hospitals, and district hospitals in 76 provinces, excluding Bangkok, serving a population of 58,921,000 people. The average population per province was 775,276, but the sizes of the provinces varied. The majority of provinces (*n* = 54, 71%) had populations between 295,920 and 1,254,640; 12 provinces (16%) had populations higher than 1,254,640 and 10 provinces (13%) had populations less than 295,920. The average ratio of doctors per 1,000 population was 0.338 (SD = 0.084). The doctor to 1000 population ratio was between 0.254 and 0.422 in the 54 middle populated provinces, more than 0.422 in the 12 highly populated provinces, and less than 0.254 in the 10 least populated provinces. The GPP per capita was categorized into quartiles: low-income provinces (55,417–80,260 Thai Baht (THB), middle-low income provinces (80,261–110,961 THB), middle-high income provinces (110,962–165,605 THB) and high-income provinces (more than 165,605 THB). The majority of the population lived in either low or middle-low income provinces (57.8%), 27.6% of the population lived in high-income provinces, and 14.5% of the population lived in middle-high income provinces. In relation to the doctor per 1000 population ratio, the densities of doctor per population were similar among all income quartiles although high-income provinces and low-income provinces had slightly lower ratios (Table 1) in 2017.

**Table 1:** Doctor to 1000 population ratio and Gross Provincial Products (GPP) of 76 provinces in 2017

GPP and population ratio		Number (%/SD)
Population (× 1000)	Average (SD)	775.276 (479.361)
	1. < 295.92 2. 295.92–775.28 3. 775.29–1254.64 4. > 1254.64	10 (13.2%) 28 (36.8%) 26 (34.2%) 12 (15.8%)
Doctor/1000 population Number (%)	Average (SD)	0.338 (0.084)
	1. < 0.253 2. 0.254–0.338 3. 0.339–0.422 4. > 0.422	10 (13.2%) 28 (36.8%) 26 (34.2%) 12 (15.8%)
GPP per capita Number (%)	Average (SD)	162,378.16 (157,538.21)
	1. 55,417–80,260 2. 80,261–110,961 3. 110,962–165,605 4. > 165,605	22 (28.9%) 22 (28.9%) 11 (14.5%) 21 (27.6%)
GPP per capita VS Doctor/1000 population Mean (SD)	1. 55,417–80,260 2. 80,261–110,961 3. 110,962–165,605 4. > 165,605	0.31 (0.06) 0.37 (0.07) 0.34 (0.07) 0.33 (0.12)

The overall supply of doctors was significantly higher in 2021, and the percentage increase in the number of doctors was higher than the increase in the population (12.5% compared to 2.9%). Provinces with the lowest doctor-to-population ratios in 2017 (< 0.253) had the highest increase in the doctor density ratio (47.1%) in 2021, but middle doctor density provinces (0.254–0.338 and 0.339–0.422) also showed percentage increases (26.1% and 13.1%, respectively), as did the provinces with the highest doctor density (> 0.422), which showed a 15.8% increase. In 2021, the percentage increases in the doctor-to-population ratios among the doctor per population ratio categories were significant (one-way ANOVA analysis (*F* = 6.94, *P* < 0.001).

In respect to doctor-to-population ratio and GPP per capita, the highest percentage increase in the doctor per population ratio was in the provinces with the highest GPP per capita (38.5%), followed by that of the middle-high income (26.4%), middle-low income (15.5%) and low-income provinces (13.4%). The percentage increases in the doctor-to-population ratios among the 4 provincial income categories were significant (*F* = 6.244, *p* < 0.001). See Table 2.

**Table 2:** One-way ANOVA to analyze the changes of doctor per 1,000 population ratio in 2017 and 2021

Doctor density and GPP	In 2017	In 2021	Change (%)	*F/P *value
Number of doctors	17,072	19,201	2129 (12.5%)	NA
Population	58,921,000	60,643,445	1,722,445 (2.9%)	NA
Doctor/1000 population				*F* = 6.94 *P* < 0.001
1. < 0.253	0.20 (0.05)	0.28 (0.05)	0.08/47.1%	
2. 0.254–0.338	0.3 (0.02)	0.38 (0.05)	0.08/26.1%	
3. 0.339–0.422	0.37 (0.24)	0.42 (0.08)	0.05/13.1%	
4. > 0.422	0.47 (0.033)	0.54 (0.33)	0.07/15.8%	
GPP per capita				*F* = 6.244 *P* < 0.001
1. 55,417–80,260	0.31 (0.06)	0.35 (0.07)	0.04/13.4%	
2. 80,261–110,961	0.37 (0.07)	0.42 (0.07)	0.05/15.5%	
3. 110,962–165,605	0.34 (0.07)	0.43 (0.13)	0.09/26.4%	
4. > 165,605	0.33 (0.12)	0.44 (0.1)	0.11/38.5%	

### Relationship between doctor density and provincial economic situation

We calculated the bivariate coefficient correlation between doctor density (represented by doctor per 1000 population) and provincial economic situation (represented by GPP) in 2017 and 2021 using the Pearson correlation coefficient. The calculated Pearson correlation coefficient values indicated no correlation in 2017 and a moderate positive correlation in 2021 between the density of doctors and GPP (*F* = 0.279, *p* = 0.018).

**Fig. 1:**
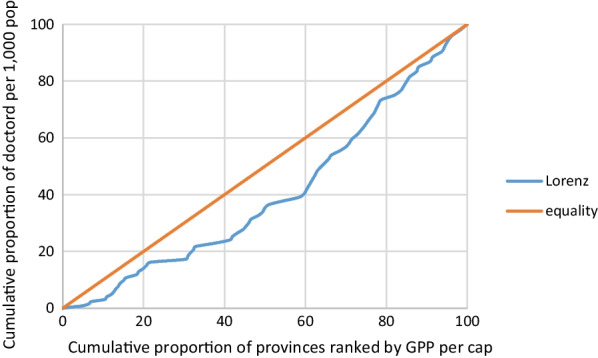
Lorenz curve of doctor density and GPP per capita at provincial level in 2017

**Fig. 2:**
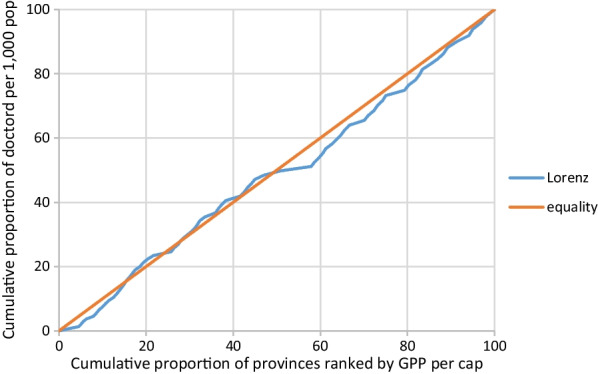
Lorenz curve of doctor density and GPP per capita at provincial level in 2021

Lorenz curves between doctor density and GPP were computed and are plotted in Figs. 1 and 2. Gini coefficients were computed to assess the degree of equitable distribution of doctors. Figure 1 shows that the Lorenz curve slightly deviates from the diagonal of the plot (which represents the equality line) and the Gini coefficient is close to 0 (0.191), indicating that the distribution of doctors across provincial incomes in 2017 was relatively equitable. The distribution of doctors in 2021 was more equitable than in 2017. The Lorenz curve for 2021 aligns closely with the equality line (Fig. 2), and the Gini coefficient for 2021 (0.03) was closer to 0. Thus, the distribution of doctors across provincial incomes improved from 2017 to 2021.

## Discussion

The current study used doctor-to-population ratios and the GPPs of 76 provinces to measure the equity of doctor distribution. Lorenz curves and the Gini coefficient were used to quantify the equity of doctor distribution. The results show that after the MoPH initiated a program to allocate newly graduated doctors according to health service utilization in 2017, the doctor distribution improved and became more equitable. The percentage changes in doctor–population ratios were highest in the provinces that had the lowest doctor densities in 2017. However, the percentage changes were also high in higher-income provinces. Overall, the Gini coefficient decreased from 0.191 in 2017 to 0.03 in 2021 indicating that the equity distribution of doctors improved.

The Gini coefficient has been used by several studies to assess geographical distribution. The results of this study are comparable to a study undertaken in Turkey that used Lorenz curves and the Gini coefficient to evaluate policies targeting more equitable distribution of health personnel and found that geographical imbalances in health personnel improved over 15 years [20]. Also, in Brazil, a government program to increase the number of doctors in remote and deprived areas showed a trend towards a more equal distribution of primary care doctors using Lorenz curves and the Gini coefficient [5].

Some studies have shown improvement in the overall distribution of doctors following the implementation of polices to improve equity while the distribution between subgroups has remained unequal, particularly between rural and urban areas and between high- and low-income areas. One study in China, found that while the spatial distribution of doctors increased overall, the Gini coefficient between rural and urban areas also increased, indicating a less equitable distribution of these doctors [19]. In Japan, another study found that the overall number of doctors increased, but the equity in geographic distribution worsened. In that study, the ratio of doctors per 100,000 population actually decreased in all areas except for urban areas when the health care demand was adjusted for changes in the population age structure [3].

Differences in the economic development situation of subgroups have also been found to contribute to the inequitable distribution of doctors. A study in Portugal found that the geographical inequality of doctor distribution was high and that this appeared to be mainly due to geographic income inequality [21]. Also, a study in Iran reported that inequality in the geographical distribution of doctors increased despite the overall number of doctors increasing as highly economically developed provinces could attract more doctors than less developed provinces [22]. It is evident that there is a significant relationship between doctor density and economic development. A study in the EU using Pearson’s correlation coefficient demonstrated a moderate positive relationship between doctor density and Gross Domestic Product (GDP) per capita, concluding that regions with high economic development (GDP per capita) had high densities of doctors [2]. This result was echoed by a study in Thailand conducted by Witthayapipobsakul et al. [4], which observed a positive relationship between doctor density and GPP, and the findings of the current study also confirm this.

The results from this study suggest that the distribution of doctors in Thailand is relatively equitable. The MoPH distributes newly graduated doctors based on the service utilization at each facility. This distribution approach appears to have improved the equity of doctor distribution. However, equitable distribution cannot be attributed solely to the distribution of newly graduated doctors, the retention and movement of existing doctors is an equally important factor. Since the 1960s, Thailand has implemented multiple strategies to retain doctors and health personnel serving in MoPH facilities [10–14]. With these strategies, doctors could be retained for longer in rural areas and in MoPH health facilities. It is rational to conclude that it is the synergy between the existing retention strategies and the newly graduated doctor distribution measures (based on service utilization) that have improved the equity distribution of doctors in Thailand.

There are some limitations to this study that should be addressed for cautious interpretation. (1) The data were aggregated at the provincial level and focused particularly on doctors working at MoPH managed facilities. The lack of data about doctors working in the private sector and in other public health facilities would underestimate the number of doctors working in each province. However, as the vast majority of facilities at provincial levels are under the control of the MoPH, the doctor numbers used in this study are likely to be a suitable proxy for the overall doctor distribution. (2) The study used snapshots of data from 2017 and 2021. The number of newly graduated doctors entering the MoPH was not available, therefore, the increased number of doctors in 2021 is the result of the interplay among newly graduated doctors entering MoPH facilities, doctors moving among facilities, and doctors leaving or resigning. (3) The provincial GPP is a proxy for economic development and might not perfectly represent household economic status. (4) The study did not cover distribution between rural and urban areas. However, a previous study conducted in Thailand by Witthayapipobsakul et al. [4] found that doctors and other health professionals were concentrated in rural areas in lower income provinces. This could possibly indicate that doctor distribution in rural areas has improved. Despite these limitations, this study provides crucial insights into the equitable distribution of doctors in Thailand.

## Conclusion

This study assessed the effects of MoPH allocation of doctors based on service utilization. The equity of doctor distribution improved from 2017 to 2021, particularly in provinces that had lower doctor densities in 2017 and in high-income provinces. The equitable distribution of doctors is affected by increased numbers of newly graduated doctors and low turnover rates of existing doctors.

## Data Availability

The data sets used and/or analyzed during the current study are available from the corresponding author on reasonable request.

## Abbreviations

GDP: Gross Domestic Product
GPP: Gross provincial products
MoPH: Ministry of Public Health
SD: Standard deviation
THB: Thai Baht
